# Social inequality in cancer survivorship: Educational differences in health‐related quality of life among 27,857 cancer survivors in Denmark

**DOI:** 10.1002/cam4.6596

**Published:** 2023-09-29

**Authors:** Anne Katrine Graudal Levinsen, Trille Kristina Kjaer, Lau Caspar Thygesen, Thomas Maltesen, Erik Jakobsen, Ismail Gögenur, Michael Borre, Peer Christiansen, Robert Zachariae, Peter Christensen, Søren Laurberg, Peter de Nully Brown, Lisbet Rosenkrantz Hölmich, Christoffer Johansen, Susanne K. Kjær, Lonneke van de Poll‐Franse, Lena Saltbæk, Susanne Oksbjerg Dalton

**Affiliations:** ^1^ Survivorship and Inequality in Cancer Danish Cancer Institute Copenhagen Denmark; ^2^ National Institute of Public Health University of Southern Denmark Copenhagen Denmark; ^3^ Statistics and Data Analysis Danish Cancer Institute Copenhagen Denmark; ^4^ Department of Thoracic surgery Odense University hospital Odense Denmark; ^5^ Dept. Surgery, Center for Surgical Science Zealand University Hospital Køge Denmark; ^6^ Institute for Clinical Medicine Copenhagen University Copenhagen Denmark; ^7^ Department of Urology Aarhus University Hospital Aarhus Denmark; ^8^ Danish Breast Cancer Group Center and Clinic for Late Effects Aarhus Denmark; ^9^ Department of Plastic and Breast Surgery Aarhus University Hospital Aarhus Denmark; ^10^ Danish Cancer Society Centre for Research on Survivorship and Late Adverse Effects After Cancer in the Pelvic Organs, Department of Surgery Aarhus University Hospital Aarhus Denmark; ^11^ Department of Hematology Rigshospitalet Copenhagen Denmark; ^12^ Department of Plastic Surgery Copenhagen University Hospital Herlev Denmark; ^13^ Cancer late effects, Rigshospitalet University of Copenhagen Copenhagen Denmark; ^14^ Unit of Virus, Lifestyle and Genes Danish Cancer Institute Copenhagen Denmark; ^15^ Department of Gynecology, Rigshospitalet University of Copenhagen Copenhagen Denmark; ^16^ Department of Psychosocial Research and Epidemiology The Netherlands Cancer Institute Amsterdam The Netherlands; ^17^ Center of Research on Psychology in Somatic diseases, Department of Medical and Clinical Psychology Tilburg University Tilburg The Netherlands; ^18^ Danish Research Center for Equality in Cancer, Department of Clinical Oncology & Palliative Care Zealand University Hospital Næstved Denmark

**Keywords:** cancer survivorship, health‐related quality of life, social inequality

## Abstract

**Background:**

With a growing population of cancer survivors in Denmark, the evaluation of health‐related quality of life (HRQoL) has become increasingly important. We describe variations in HRQoL between educational groups in a national population of cancer survivors.

**Methods:**

We conducted a cross‐sectional questionnaire study among breast, prostate, lung, and colon cancer survivors diagnosed in 2010–2019 in Denmark. We used the EORTC QLQ‐C30 to assess HRQoL including physical, role, emotional, cognitive, social functioning, and symptoms (fatigue, nausea and vomiting, pain, dyspnea, insomnia, appetite loss, constipation, diarrhea, and financial difficulties). Information on educational level and clinical data were extracted from national registers and clinical databases. Levels of impaired functioning and severe symptoms were identified using newly established thresholds for clinical importance. Multivariate logistic regression was used to examine associations between education and HRQoL. All statistical tests were 2‐sided.

**Results:**

In total, 27,857 (42%) participated in the study. Up to 72% and 75% of cancer survivors with short education (≤9 years) reported impaired functioning and severe symptoms, respectively. Cancer survivors with short compared to long education (>12 years) were more likely to report impaired functioning and severe symptoms, with for example significantly higher odds ratios (ORs) for impaired physical function (breast OR = 2.41, 99% CI = 2.01–2.89; prostate OR = 1.81, 99% CI = 1.48–2.21; lung OR = 2.97, 99% CI = 1.95–4.57; and colon cancer OR = 1.69, 99% CI = 1.28–2.24).

**Conclusions:**

Cancer survivors with short education are at greater risk of impaired HRQoL than survivors with long education 2–12 years after diagnosis. This underscores the need for systematic screening and symptom management in cancer aftercare, in order to reach all cancer survivors, also cancer survivors with short education.

## 1 BACKGROUND

Improved diagnostic detection and advances in cancer treatment have led to a growing population of cancer survivors.^1^, ^2^, ^3^ However, many cancer survivors develop a range of physical and mental late effects,^2^, ^4^, ^5^, ^6^, ^7^ and report poorer health‐related quality of life (HRQoL) than a general population.^8^, ^9^ Previous research among selected populations of cancer survivors suggest that comorbidity,^8^, ^10^, ^11^ obesity, smoking,^11^ physical activity,^12^ and other lifestyle factors,^9^ social support,^13^ and individual resilience^14^ are associated with poor HRQoL in cancer survivors. In addition to these classical risk factors for poor HRQoL, some studies have suggested that socioeconomic factors, e.g., living in a disadvantaged area,^15^, ^16^ or having a low socioeconomic position (SEP)^8^, ^9^, ^11^ are associated with poorer HRQoL but these are limited by few participants^8^, ^9^ and non‐cancer specific measures of HRQoL.^11^, ^16^ Only one of these studies investigated the association between SEP and HRQoL in a nationally representative sample of cancer survivors but the study only included 2235 survivors after breast cancer.^15^ We need large population‐based data to explore the association between SEP indicators such as educational level and HRQoL among cancer survivors across major cancer sites, for both short‐and long‐terms survivors. Evaluation of social inequality in HRQoL after cancer will allow us to identify cancer survivors at higher risk of adverse HRQoL outcomes. The clinical challenge is how to assign the limited healthcare resources, and by identifying the most vulnerable cancer survivors we can offer personalized follow‐up and systematic screening of symptoms in cancer aftercare for those who are most in need.

In this large‐scale population‐based study, we combined information from questionnaires, national registration of educational level, and information from clinical databases in more than 25,000 cancer survivors diagnosed between 2010 and 2019 with the most prevalent cancers in Denmark: breast, prostate, lung, and colon cancer.^17^ We applied recently published thresholds for clinically important impairments in the HRQoL domains of functioning and symptoms to identify cancer survivors who reported functioning and symptoms at levels assumed to require attention from a healthcare professional.^18^

## 2 METHODS

### 2.1 Study participants and study design

Survivors after breast, prostate, lung, and colon cancer were identified in the Danish Cancer Registry.^19^ Eligible participants had to be diagnosed between January 1st, 2010 and December 31st, 2019, be 40 years or older at the time of diagnosis, and be residents in Denmark. Since 1968, all residents in Denmark are provided with a unique identification number (CPR number), which allows accurate individual‐level linkage across national registers.^20^ We used the CPR number to link our questionnaire data with data from the Danish nationwide health and administrative registers,^20^ and with information from the clinical databases as outlined below.^21^, ^22^, ^23^, ^24^

All eligible survivors were invited to participate in the study through e‐Boks, the Danish national secure digital mail system, used by 93.3% of the Danish population.^25^ Survivors were asked to not participate if they were in active treatment. Non‐responders received one reminder. Questionnaire data were collected between January 13th and March 14th, 2022, and all participants provided informed consent. The study was approved by the National Board of Health Data (Region Zealand (REG‐059‐2021)).

### 2.2 Questionnaire data

Participants completed validated self‐reported instruments together with study‐specific items on common comorbid disorders, physical, and lifestyle factors. For a list of included comorbid conditions and categorization (presence of 0, 1, or 2+ comorbid disorders), categorization of lifestyle, and anthropometric factors, see Table S1.

To assess their HRQoL, participants completed the European Organization for Research and Treatment of Cancer Quality of Life Core Questionnaire (EORTC QLQ‐C30).^26^ The EORTC QLQ‐C30 consists of 30 items covering one global quality of life scale, five functioning scales (physical, comprised of the domains strenuous activities, short and long walks, need to sit/lay down, help dressing, eating, and washing), role (domains: work and household jobs, cognitive concentrating, reading, watching television, and remembering), emotional (domains: feel tense, worried, irritable, and depressed), and social functioning (domains: family life and social activities). The questionnaire also includes three symptom scales: fatigue (domains: need for rest, feeling weak, and tired), pain (domains: pain, pain interfering with daily activities), and nausea and vomiting (domains: felt nauseated, vomited), and six single item symptoms: dyspnea, insomnia, appetite loss, constipation, diarrhea, and financial difficulties. All scales and single items were scored from 0 to 100 according to the EORTC guidelines.^27^ To identify cases with impaired functioning and severe symptoms, we dichotomized each scale and single item using recently established thresholds for clinical importance as cutoff values.^18^ Giesinger et al. established these thresholds for the functioning and symptoms included in the EORTC QLQ‐C30 based on interviews and questionnaires, wherein patients could anchor each question to a level of importance (symptom/problem limits my daily life, I need help or care because of my symptom/problem or my symptom/problem causes my partner or I to worry), and statistical analysis were conducted to determine the diagnostic accuracy of each threshold.^18^

### 2.3 Registry based information

We used educational level as an indicator for SEP. Education is a strong indicator of SEP, as it is relatively stable over the adult life course, reflects a person's attained knowledge and skills and may be a proxy for other SEP indicators such as income or occupation.^28^ Furthermore, education also reflect an individual's cognitive function and understanding of health information, which may translate into better communication skills and improved access to health services.^28^

We obtained individual‐level information on education, cohabitation status, and urbanicity from the social registers administered by statistics Denmark.^20^ Information on education was measured 2 years prior to cancer diagnosis and categorized as short (mandatory school; ≤9 years), medium (secondary school/high school or vocational education; 10–12 years), and long education (higher education; >12 years). Information on cohabitation status was categorized as living with others or living alone and were measured prior to diagnosis. Information on urbanicity was measured at time of diagnosis and categorized into three area types for place of residence: cities (densely populated area), towns and suburbs (intermediate density area), and rural (thinly populated area).

### 2.4 Clinical databases

We obtained information on cancer diagnosis, stage, and treatment in the first 12 months after the cancer diagnosis from the relevant national clinical quality cancer databases: Danish Breast Cancer Cooperative Group Database,^21^ Danish Prostate Cancer Database,^22^ Danish Lung Cancer Registry,^23^ and Danish Colorectal Cancer Group Database.^24^ Stage was categorized as local/regional stage or advanced stage disease and treatment as curatively intended or palliative treatment (for categorization of disease stage and treatment for each cancer type, see Table S1).

### 2.5 Statistical analyses

Although this is a cross‐sectional study, we used directed acyclic graphs (DAGs)^29^, ^30^ to identify potential confounders and mediators on the association between education and HRQoL, as information on our exposure, educational level, was collected prior to the outcome (Figure S1). We identified age and sex as potential confounders between education and HRQoL in the DAG. All analyses were done separately for each cancer type. We identified the proportion of cancer survivors who reported clinically relevant impaired functioning and symptom severity by educational level.^18^ Logistic regression models were used to compute the association between education and HRQoL adjusted for age, sex, and time since diagnosis. In secondary analyses, we separated the analyses by survival time into 2–5 years (short‐term survivors) versus >5–12 years (long‐term survivors). We identified stage at diagnosis, treatment, urbanicity, comorbidity, body mass index (BMI), alcohol intake, smoking status, and cohabitation as potential mediators between education and HRQoL in our DAG. A counterfactual‐based mediation analyses were, however, not possible with the available cross‐sectional data. Instead, we explored if these clinical and lifestyle factors modified the association between education and HRQoL in logistic regression analyses with adjustment for the identified mediators. If the estimated effect of educational level on HRQoL decreased after mediator adjustment, we interpreted the factors (stage at diagnosis, treatment, urbanicity, comorbidity, BMI, alcohol intake, smoking status, and cohabitation) as partial mediators.^31^ To compensate for multiple testing, we used the 99% confidence interval in all regression analyses. To account for potential selection bias, we performed sensitivity analyses, reproducing the logistic regression analyses with inverse probability weighting,^32^ which adds a weight to those who were under‐ or overrepresented in our study population using measured variables (age, sex, time since diagnosis, and education). All analyses were conducted in R (version 4.1.2).^33^

## 3 RESULTS

### 3.1 Study participants

**FIGURE 1:**
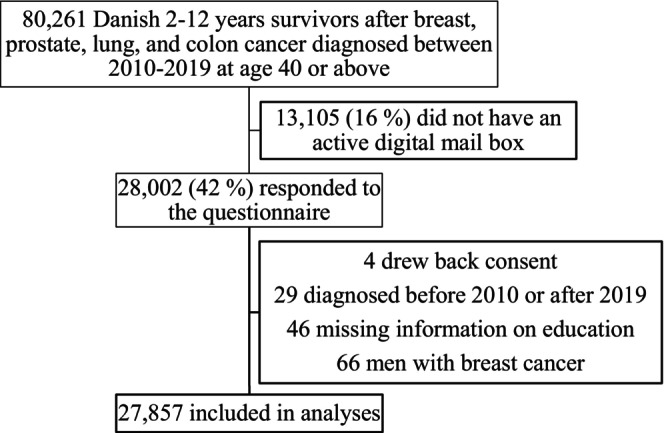
Population flowchart over the SEQUEL study including 2–12‐year Danish survivors of breast, prostate, lung, and colon cancer.

Out of the total sample of 80,261 survivors, 16% did not have an active digital e‐Boks and were thus not invited. Of the 67,156 cancer survivors who were invited to participate in the questionnaire study, 27,998 (42%) responded, with 27,857 (99.5%) fulfilling the inclusion criteria (Figure 1).

Survivors who did not have an active digital mail were on average 73 years at diagnosis and 42% had a short education (Table S2). Non‐respondents were older (mean age of 65 years vs. 63 years at diagnosis) and were more likely than respondents to have a short education (18% vs. 11%) while non‐respondents did not differ from respondents by cancer type (Table S2). Participants' age ranged from a mean of 59 years at diagnosis (breast cancer) to a mean of 67 years (prostate cancer). The majority of participants were diagnosed with local/regional stage disease, received primary curative intended treatment, and had medium education. Only 9%–12% had short education. Between 35% and 50% of participants had ≥2 comorbidities, 54%–88% were former or current smokers, 18%–21% drank more alcohol than recommended, and 19%–23% were obese (BMI ≥30) at time of questionnaire (Table 1).

**TABLE 1:** Characteristics of 27,857 Danish breast, prostate, lung, and colon cancer survivors diagnosed between 2010 and 2019.

Characteristics	Cancer survivors			
	Breast *n*=11,868 *n* (%)	Prostate *n*=9606 *n* (%)	Lung *n*=1754 *n* (%)	Colon *n*=4629 *n* (%)
Age (years): mean (range)	59 (40–93)	67 (40–89)	65 (40–91)	66 (40–92)
Sex				
Men	–	9566 (100)	753 (43)	2485 (54)
Women	11,848 (100)	–	1000 (57)	2135 (46)
Education^a^				
Short	1083 (9)	1088 (11)	284 (17)	568 (12)
Medium	5592 (47)	4920 (51)	1007 (57)	2437 (53)
Long	5193 (44)	3598 (38)	463 (26)	1624 (35)
Cohabitation^b^				
Living with others	8707 (73)	8149 (85)	1275 (73)	3562 (77)
Living alone	3141 (27)	1417 (15)	^h^ (–)	1058 (22)
Missing	20 (0)	40 (0)	^h^ (–)	9 (0)
Urbanicity^c^				
Cities	3292 (28)	2313 (24)	^h^ (–)	1196 (26)
Towns and suburbs	4358 (37)	3391 (35)	621 (35)	1623 (35)
Rural	4198 (35)	3862 (40)	679 (39)	1801 (39)
Missing	20 (0)	40 (1)	^h^ (–)	9 (0)
Time since diagnosis				
2–5 years	4269 (36)	3524 (37)	967 (55)	1882 (41)
>5–12 years	7599 (64)	6082 (63)	787 (45)	2747 (59)
Disease stage at diagnosis^d^				
Local/regional	9188 (77)	6734 (70)	884 (50)	3215 (69)
Advanced	90 (1)	364 (4)	436 (25)	266 (6)
Missing^i^	2590 (22)	2508 (26)	434 (25)	1148 (25)
Treatment during 12 month after diagnosis^e^				
Curatively intended	9188 (77)	5793 (60)	1171 (67)	3753 (81)
Palliative	90 (1)	1205 (13)	156 (9)	51 (1)
None	816 (7)	2541 (26)	28 (1)	39 (1)
Missing^j^	1774 (15)	67 (1)	399 (23)	786 (17)
Comorbidity at time of questionnaire^f^				
0	4568 (39)	3005 (31)	484 (28)	1602 (35)
1	3134 (26)	2581 (27)	385 (22)	1164 (25)
≥ 2	4166 (35)	4020 (42)	885 (50)	1863 (40)
Smoking status at time of questionnaire				
Never	5264 (44)	3790 (39)	171 (10)	1772 (38)
Former smoker	5187 (44)	4918 (51)	1289 (73)	2362 (51)
Current smoker	1234 (10)	748 (8)	261 (15)	402 (9)
Missing	183 (2)	150 (2)	33 (2)	93 (2)
Alcohol intake at time of questionnaire^g^				
0 units per week	3229 (27)	1324 (14)	543 (31)	980 (21)
≥1 to ≤7/14 units per week	5572 (47)	5897 (61)	741 (42)	2359 (51)
≥8/15 units per week	2195 (19)	1735 (18)	362 (21)	955 (21)
Missing	872 (7)	650 (7)	108 (6)	335 (7)
BMI at time of questionnaire				
Underweight (<18.5)	182 (2)	23 (0)	48 (3)	69 (2)
Normal weight (18.5–24.9)	5174 (44)	3014 (31)	711 (41)	1550 (33)
Overweight (25–29.9)	4037 (34)	4610 (48)	612 (35)	1868 (40)
Obese (≥30)	2287 (19)	1789 (19)	351 (20)	1056 (23)
Missing	188 (2)	170 (2)	32 (2)	86 (2)

Abbreviation: BMI, body mass index. [Correction added on October 5, 2023 after first online publication. In Table 1, the BMI for “Obese” has been corrected in this version.]

^a^ Education: categorized as short (mandatory school; ≤ 9 years), medium (secondary education or vocational education; 10–12 years), and long education (higher education; >12 years).

^b^ Cohabitation: living with others included all who were married, in a registered partnership or co‐living with a partner. Living alone included all who were single or widows and had no kids living at home.

^c^ Urbanicity: cities (densely populated area), towns and suburbs (intermediate density area), and rural (thinly populated area).

^d^ Disease stage: local/regional stage defined as any tumor size, any number of positive lymph nodes and no distant metastasis (breast cancer); TNM stage with any T, any N, and no M (prostate cancer); TNM stage at IA, IB, IIA, IIB, or IIIA (lung cancer), UICC (8th edition) stage I, II, III (colon cancer). Advanced stage defined as distant metastasis (breast cancer); TNM stage with metastasis (prostate cancer); TNM stage IIIB, IIIC, IVA, and IVB (lung cancer); UICC (8th edition) stage IV (colon cancer).

^e^ Treatment: curative treatment defined as local/regional stage disease (breast cancer); prostatectomy, active surveillance, curative radiotherapy (prostate cancer); curative chemo‐ and/or radiotherapy, surgery, and neo‐ and/or adjuvant therapy (lung cancer); surgery with curative intent (colon cancer). Palliative treatment defined as advanced stage disease (breast cancer); palliative radiotherapy, endocrine therapy, and watchful waiting (prostate cancer); palliative chemo‐ and/or radiotherapy, other treatment with palliative intent (lung cancer); surgery with palliative intent, chemo‐ and/or radiotherapy (colon cancer).

^f^ Comorbidity was self‐reported and included the following comorbid disorders: depression, anxiety, asthma, apoplexy, hypertension, migraine, arthritis, thyroid disorders, cardiovascular disease, osteoporosis, diabetes, impaired hearing, low vision/blindness, chronic obstructive pulmonary disease, and disorders of the nervous system (Parkinson, sclerosis).

^g^ Alcohol: defined as intake during the last week. Categorized as no alcohol intake (0 units), alcohol intake within the recommended amount by the Danish health authorities at time of questionnaire (1–7/14 units for women/men) and alcohol intake higher than the recommended amount (≥8/15 units for women/men).

^h^ All data on cohabitation and urbanicity for lung cancer survivors cannot be included in the table, as they present information on too few individuals (less than 5).

^i^ There were no clinical data available for 2019, which led to a high number of missing data for stage: 13% for breast cancer, 4% for prostate cancer, 22% for lung cancer, and 13% for colon cancer.

^j^ There were no clinical data available for 2019, which led to a high number of missing data for treatment: 13% for breast cancer, 0% for prostate cancer, 22% for lung cancer, and 13% for colon cancer.

### 3.2 Impaired functioning and severe symptoms

**FIGURE 2:**
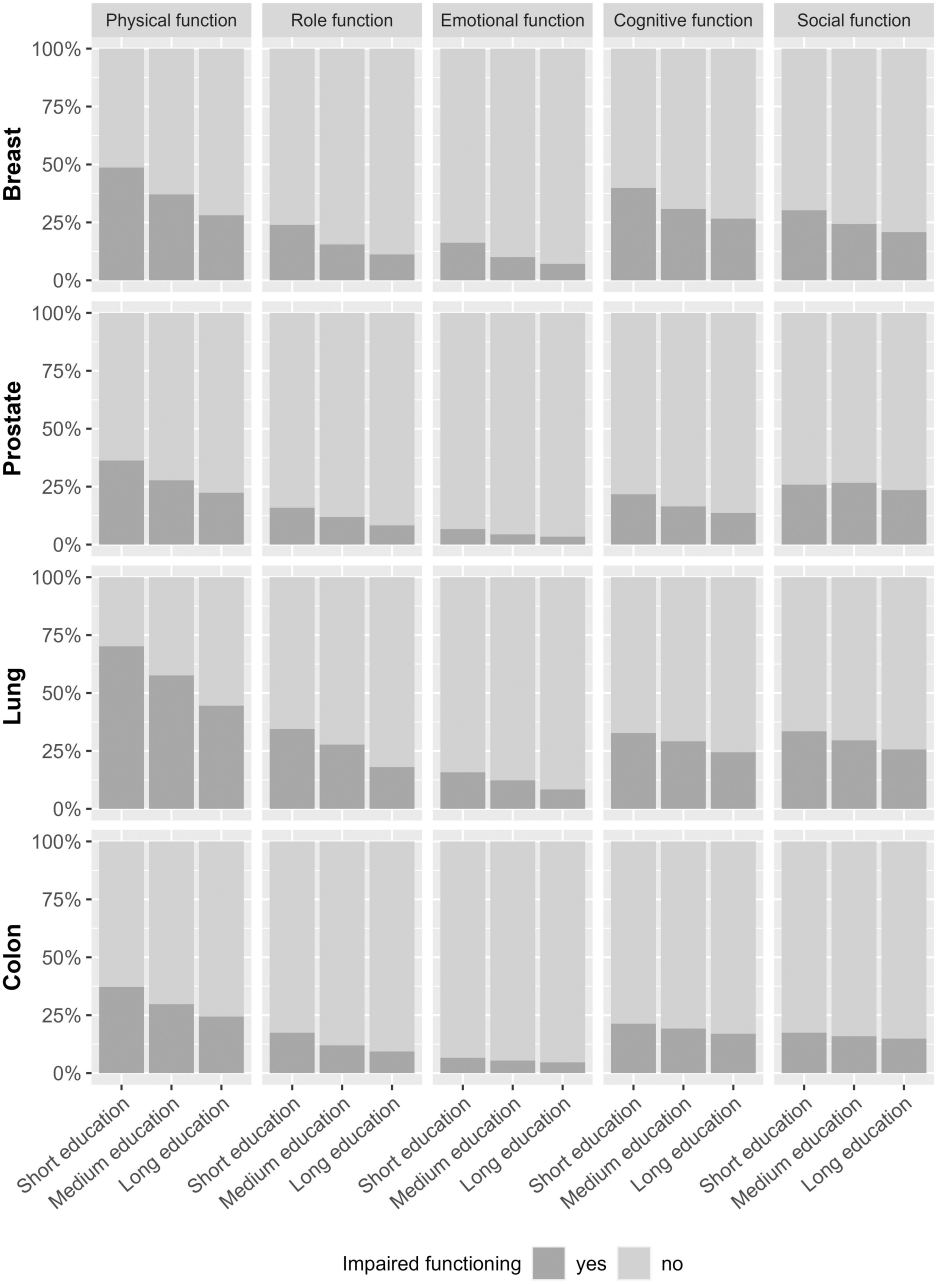
Prevalence of Danish cancer survivors who report impaired functioning by education.

**FIGURE 3:**
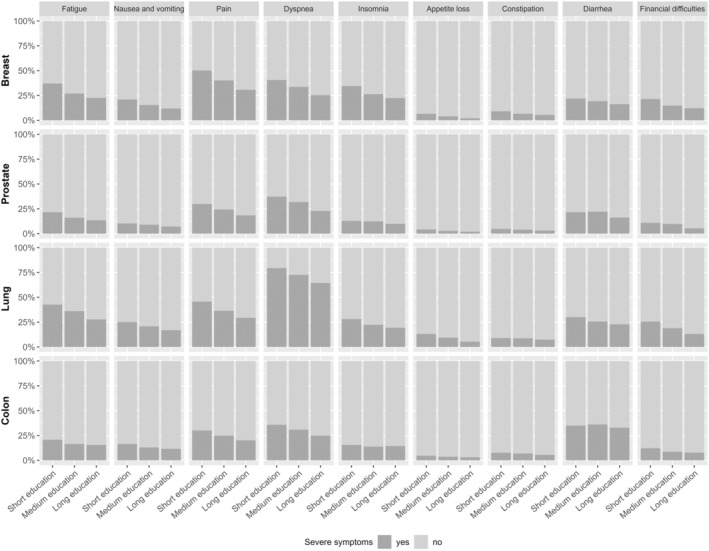
Prevalence of Danish cancer survivors who report severe symptoms by education.

The prevalence of impaired functioning and severe symptoms were higher among participants who were diagnosed with lung or breast cancer than prostate or colon cancer. Nevertheless, a higher prevalence of participants with short education reported impaired functioning and severe symptoms compared to those with long education, regardless of cancer type (Figures 2 and 3).

In the adjusted regression analyses, participants with short education were generally more likely to report impaired functioning and severe symptoms; however, with several odds ratios (ORs) not reaching statistical significance. Participants with short education from breast (OR = 2.41, 99% CI = 2.01–2.89), prostate (OR = 1.81, 99% CI = 1.48–2.21), lung (OR = 2.97, 99% CI = 1.95–4.57), and colon cancer (OR = 1.69, 99% CI = 1.28–2.24) were more likely to report impaired physical functioning compared with participants with long education (Table 2). Similarly, participants with short education from breast (OR = 2.18, 99% CI = 1.80–2.64), prostate, (OR = 1.70, 99% CI = 1.34–2.15), lung (OR = 1.95, 99% CI = 1.28–2.99), and colon cancer (OR = 1.41, 99% CI = 1.01–1.96) were more likely to report severe fatigue, pain (breast OR = 2.37, 99% CI = 1.98–2.85; prostate OR = 1.87, 99% CI = 1.51–2.30; lung OR = 2.07, 99% CI = 1.36–3.15; and colon cancer OR = 1.72, 99% CI = 1.28–2.31), and dyspnea (breast OR = 1.99, 99% CI = 1.65–2.39; prostate OR = 1.92, 99% CI = 1.58–2.34; lung OR = 2.14, 99% CI = 1.35–3.44; and colon cancer OR = 1.63, 99% CI = 1.23–2.16) compared with participants with long education (Table 2). Although ORs were less increased and not all reached statistical significance, participants with medium education were also more likely to experience impaired functioning and severe symptoms compared to participants with long education (Table 2). Estimates from sensitivity analyses were consistent with these findings (results not presented).

**TABLE 2:** The association between educational level and the risk of reporting impaired functioning or symptoms at clinically important levels in Danish breast, prostate, lung, and colon cancer survivors.

	Education^a^	Cancer survivors			
		Breast	Prostate	Lung	Colon
		OR (99% CI)	OR (99% CI)	OR (99% CI)	OR (99% CI)
Functioning scales (EORTC QLQ‐C30)					
Physical function	Short	2.41 (2.01; 2.89)	1.81 (1.48; 2.21)	2.97 (1.95; 4.57)	1.69 (1.28; 2.24)
	Medium	1.50 (1.34; 1.67)	1.37 (1.20; 1.57)	1.69 (1.25; 2.28)	1.26 (1.04; 1.53)
Role function	Short	2.58 (2.06; 3.22)	1.98 (1.50; 2.60)	2.40 (1.52; 3.81)	1.95 (1.34; 2.83)
	Medium	1.50 (1.29; 1.74)	1.51 (1.25; 1.85)	1.75 (1.22; 2.55)	1.29 (0.98; 1.71)
Emotional function	Short	2.71 (2.07; 3.52)	2.16 (1.43; 3.21)	2.08 (1.11; 3.91)	1.53 (0.86; 2.64)
	Medium	1.55 (1.28; 1.86)	1.30 (0.96; 1.76)	1.63 (0.99; 2.79)	1.20 (0.82; 1.79)
Cognitive function	Short	2.10 (1.73; 2.55)	1.75 (1.38; 2.20)	1.53 (0.97; 2.39)	1.39 (1.00; 1.92)
	Medium	1.37 (1.22; 1.54)	1.25 (1.06; 1.47)	1.34 (0.95; 1.90)	1.19 (0.96; 1.49)
Social function	Short	1.81 (1.48; 2.22)	1.14 (0.92; 1.41)	1.47 (0.94; 2.29)	1.26 (0.88; 1.78)
	Medium	1.33 (1.17; 1.50)	1.18 (1.03; 1.35)	1.26 (0.90; 1.78)	1.11 (0.88; 1.40)
Symptom scales and single items (EORTC QLQ‐C30)					
Fatigue	Short	2.18 (1.80; 2.64)	1.70 (1.34; 2.15)	1.95 (1.28; 2.99)	1.41 (1.01; 1.96)
	Medium	1.34 (1.19; 1.51)	1.24 (1.05; 1.46)	1.51 (1.09; 2.10)	1.07 (0.85; 1.35)
Nausea/vomiting	Short	1.99 (1.58; 2.49)	1.51 (1.10; 2.06)	1.65 (1.01; 2.71)	1.56 (1.07; 2.24)
	Medium	1.37 (1.18; 1.59)	1.28 (1.03; 1.59)	1.32 (0.90; 1.97)	1.15 (0.89; 1.49)
Pain	Short	2.37 (1.98; 2.85)	1.87 (1.51; 2.30)	2.07 (1.36; 3.15)	1.72 (1.28; 2.31)
	Medium	1.57 (1.41; 1.74)	1.43 (1.24; 1.64)	1.43 (1.04; 1.98)	1.32 (1.07; 1.62)
Dyspnea	Short	1.99 (1.65; 2.39)	1.92 (1.58; 2.34)	2.14 (1.35; 3.44)	1.63 (1.23; 2.16)
	Medium	1.48 (1.32; 1.65)	1.59 (1.39; 1.82)	1.46 (1.06; 2.00)	1.33 (1.10; 1.61)
Insomnia	Short	1.91 (1.57; 2.31)	1.34 (1.01; 1.78)	1.65 (1.03; 2.66)	1.11 (0.76; 1.58)
	Medium	1.28 (1.14; 1.45)	1.28 (1.07; 1.55)	1.25 (0.86; 1.82)	0.96 (0.75; 1.22)
Appetite loss	Short	3.28 (2.15; 4.96)	2.05 (1.21; 3.38)	2.62 (1.29; 5.47)	1.48 (0.75; 2.82)
	Medium	1.93 (1.41; 2.65)	1.44 (0.99; 2.13)	1.85 (1.03; 3.54)	1.19 (0.75; 1.93)
Constipation	Short	1.75 (1.26; 2.41)	1.41 (0.88; 2.21)	1.22 (0.58; 2.51)	1.41 (0.83; 2.32)
	Medium	1.23 (0.99; 1.52)	1.34 (0.98; 1.84)	1.21 (0.71; 2.15)	1.26 (0.89; 1.80)
Diarrhea	Short	1.44 (1.15; 1.79)	1.42 (1.12; 1.78)	1.45 (0.92; 2.28)	1.10 (0.83; 1.45)
	Medium	1.22 (1.07; 1.39)	1.47 (1.27; 1.71)	1.17 (0.83; 1.66)	1.15 (0.97; 1.38)
Financial difficulties	Short	2.24 (1.77; 2.83)	2.34 (1.68; 3.23)	2.39 (1.40; 4.09)	1.86 (1.20; 2.84)
	Medium	1.41 (1.21; 1.64)	1.89 (1.50; 2.39)	1.71 (1.12; 2.68)	1.19 (0.87; 1.63)

*Note*: Long education is the reference for all analyses. All analyses are adjusted for age at diagnosis, sex, and time since diagnosis.

Abbreviations: CI, confidence interval; EORTC QLQ‐C30, European Organization for Research and Treatment of Cancer Quality of Life Core Questionnaire; OR, odds ratio.

^a^ Education: categorized as short (mandatory school; ≤9 years), medium (secondary education or vocational education; 10–12 years), and long education (higher education; >12 years).

Separate analyses stratified by time since diagnosis indicated that ORs for impaired functioning and severe symptoms were increased for participants with medium or short education compared to participants with long education. This was found for both short‐ and long‐term survivors, although most estimates for long‐term survivors failed to reach statistical significance (Table S3).

The mediator‐adjusted ORs for impaired HRQoL changed slightly towards the null for all functioning scales and symptoms; however, ORs remained increased for survivors with short compared to long education. Cancer survivors with short compared to long education still had statistically significantly increased ORs for physical functioning, role functioning, pain, and financial difficulties, except among survivors after colon cancer (Table S4).

## 4 DISCUSSION

The present cross‐sectional study on educational differences in HRQoL among survivors after breast, prostate, lung, and colon cancer is the largest to date. It includes a nationwide population‐based sample of survivors spanning from 2 to 12 years after the cancer diagnosis, with self‐reported and registry linkage individual‐level data on education, clinical characteristics, treatment, comorbidity, lifestyle factors, and HRQoL domains of functioning and symptoms. About a third of all participating cancer survivors reported impaired functioning and severe symptoms. Regardless of cancer type, survivors with short education were at higher risk of experiencing impaired functioning and severe symptoms compared to survivors with long education, and these educational disparities in HRQoL persisted up to 12 years after diagnosis.

Consistent with results of previous studies, we found shorter education to be associated with an increased risk of impaired physical function, fatigue, and pain in cancer survivors.^8^, ^9^, ^11^, ^15^, ^16^ For example, one study among 2235 breast cancer survivors reported that survivors from a lower social class had an OR of 1.7 to report poor physical function.^15^ Another study of 301 colorectal cancer survivors found that those with low income were more likely to report higher levels of pain and fatigue than high income survivors.^8^ A third study of 2115 cancer survivors of mixed cancer types found SEP to be positively correlated with HRQoL.^16^ The limitations of these earlier studies include using area‐based^16^ or self‐reported^8^, ^15^ measures of SEP, using non‐cancer specific HRQoL measures (EQ‐5D‐5L),^16^ and not including thresholds for clinical important levels of HRQoL.^8^, ^9^, ^11^, ^15^, ^16^

There is a need to uncover the mechanisms driving this socioeconomic inequality in HRQoL. Differences in lifestyle and health behaviors may drive some of the associations with educational disparity. A study on socioeconomic disparities in health behaviors show that people with low SEP are more likely to engage in behaviors which are detrimental for health, such as poor dietary habits and that low SEP is associated with an almost three times higher risk of smoking and not exercising.^34^ Overall, in our study, the observed educational disparities in the risk of impaired functioning and severe symptoms remained considerable after adjustment for several clinical and lifestyle characteristics, such as stage, comorbidity, BMI, alcohol intake and smoking status. However, other factors such as health literacy,^35^ communication between cancer survivors and healthcare professionals,^36^, ^37^ and adherence to treatment^38^, ^39^ also differ among cancer survivors with different educational levels and could be potential drivers of the observed associations. A study show that shorter education is associated with lower health literacy and self‐assessed health.^35^ Hence, an educational difference in health literacy might be a potential driver of the educational differences in reported functioning levels and symptom severity among cancer survivors observed in the present study, as health literacy is associated with healthcare communication and healthcare seeking behavior. It is of vital importance that healthcare professionals are aware that survivors have different capabilities in understanding their symptoms and how to seek help and adhere to treatment within the healthcare system.

It is well‐known that long‐term survivors experience substantial fatigue and pain,^40^ but dyspnea has to our knowledge not previously been reported as a severe symptom experienced by cancer survivors. One explanation could be our use of the relatively new thresholds for clinical importance,^18^ as previous studies that have dichotomized functioning and symptoms measured with the QLQ‐C30 commonly set cutoff values below 66 and above 33, respectively.^41^, ^42^ In contrast, the cutoff for dyspnea in the present study was >17.^18^ This new cutoff may provide a better indication of the clinically important level of dyspnea, as it has been validated and the diagnostic accuracy tested.^18^, ^43^, ^44^ Poorly controlled fatigue, pain, and dyspnea has been shown to be a principal driver of preventable emergency department visits,^45^, ^46^ and our study finds a social inequality in who may be at risk for experiencing these symptoms, irrespective of cancer type. An optimization of the continuity of care for vulnerable cancer survivors and programs targeted at improving cancer survivors' level of functioning and diminishing symptoms is needed. Although there are cancer survivors with similar needs in all educational groups, a greater proportion of cancer survivors with short education are affected by high levels of symptoms and impaired functioning. An increased awareness of such socioeconomic differences in HRQoL between cancer survivors in the clinical practice in follow‐up care and beyond is called for, including systematic screening and management of symptoms affecting everyday life. Systematic symptom screening and use of patient centered tools like shared decision making may be applied, also in oncological follow‐up programs. The organizational structures of communication and navigation between i.e., hospital setting and general practice for cancer survivors should also be strengthened and accessible for alle survivors, not just those who have resources to navigate themselves, or by the help of relatives. Even though the prevalence, and risk, of poor HRQoL differed by educational level, a systematic screening and management of impaired functioning and severe symptoms in follow‐up care for all cancer survivors may enhance HRQoL overall, and thereby diminish the inequality.

### 4.1 Strengths and limitations

The strengths of the present study include a large national sample of short‐ and long‐term cancer survivors of common cancer types, thus strengthening the generalizability of our findings, the use of information on stage of disease, treatment, and education from national registers, the use of a validated HRQoL measure,^47^ and the use of validated cutoffs tailored to each of the five assessed functioning scales and nine symptoms, thereby improving the clinical relevance of our findings.^18^ Another strength is that Denmark has a universal healthcare system, which provides free and equal access to treatment for all residents.^48^ Healthcare is centralized into five regions, and there are national guidelines for cancer treatment and follow‐up care. In principle, Danish cancer survivors should be offered the same treatments and follow‐up services no matter of the location of their residence although local differences may occur. We adjusted for urbanicity in sub‐analyses, without any considerable changes to the observed educational differences in HRQoL.

Some limitations should also be noted. Our data on comorbidity, physical, and lifestyle factors relied on self‐report and could be influenced by information bias. The results could also be affected by selection bias, i.e., by factors associated with being more likely to participate in the questionnaire study, e.g., age or education. The response proportion under 50% suggested that caution is needed when interpreting results; therefore, we employed inverse probability weighting^32^ to incorporate adjustment for non‐response to our questionnaire based on differences in age, sex, and educational level in sensitivity analyses. These weighted analyses showed estimates consistent with our main results, supporting the generalizability of our findings. Still, cancer survivors with short education were underrepresented in our sample, and non‐respondents may differ from respondents on parameters we have not assessed.^49^ Cancer recurrence or progression could affect participation, and participants with recurrence or progression are likely to report worse HRQoL than participants without. This could lead to impaired functioning and severe symptoms among cancer survivors being overreported, although we did ask cancer survivors not to participate in the questionnaire study if they received active treatment. Still, patients with undiagnosed recurrence may have participated, and this could have influenced their outcome scores. It may be that patients with shorter educational levels are slightly overrepresented among such a group of patients with undiagnosed relapse. Even though we consider the use of valid and reliable measures of HRQoL as a strength, the subjectivity of symptom report among cancer survivors may have biased our findings. We included four different cancer types, which limits the generalizability of our findings, still they represent the most prevalent cancers in Denmark, and reflect diverse patient populations in terms of gender, age, treatment, and prognosis.

### 4.2 Conclusion

In this nationwide study among 27,857 survivors after breast, prostate, lung, and colon cancer, educational inequalities were apparent with survivors with short or medium education being at higher risk of HRQoL impairment across all studied cancer types, persisting up to 12 years after the cancer diagnosis. To address this inequality in cancer survivorship, there is a need to target efforts to improve HRQoL, for example through systematic screening of impaired functioning and severe symptoms during cancer follow‐up schemes, to ensure identification of survivors in need, to improve communication between survivors and clinicians, and to better manage survivors' functioning and symptoms, as poor symptom control may lead to greater symptom severity and reduced HRQoL.

## AUTHOR CONTRIBUTIONS

**Anne Katrine Graudal Levinsen:** Conceptualization (equal); formal analysis (equal); investigation (equal); methodology (equal); project administration (equal); writing – original draft (lead). **Trille Kristina Kjaer:** Conceptualization (equal); investigation (equal); methodology (equal); project administration (equal); supervision (equal); writing – review and editing (equal). **Lau Caspar Thygesen:** Supervision (equal); writing – review and editing (equal). **Thomas Maltesen:** Formal analysis (supporting); writing – review and editing (equal). **Erik Jakobsen:** Writing – review and editing (equal). **Ismail Gögenur:** Writing – review and editing (equal). **Michael Borre:** Writing – review and editing (equal). **Peer Christiansen:** Writing – review and editing (equal). **Robert Zachariae:** Writing – review and editing (equal). **Peter Christensen:** Writing – review and editing (equal). **Søren Laurberg:** Writing – review and editing (equal). **Peter Brown:** Writing – review and editing (equal). **Lisbet Hoelmich:** Writing – review and editing (equal). **Christoffer Johansen:** Writing – review and editing (equal). **Susanne K. Kjær:** Writing – review and editing (equal). **Lonneke van de Poll‐Franse:** Writing – review and editing (equal). **Lena Saltbæk:** Writing – review and editing (equal). **Susanne Oksbjerg Dalton:** Conceptualization (equal); funding acquisition (equal); investigation (equal); methodology (equal); project administration (equal); supervision (equal); writing – review and editing (equal).

## FUNDING INFORMATION

This work was supported by the Novo Nordisk Foundation (number NNF18OC0052543); the Danish Cancer Society “Videnskabelige Udvalg” (number R269‐A15811); and Helsefonden (number 20‐B‐0434).

## CONFLICT OF INTEREST STATEMENT

The authors (AKGL, TKK, LCT, TM, EJ, IG, MB, PC, RZ, PC, SL, PB, LRH, CJ, SKK, LvdPF, LS, SOD) declare no competing interests.

## ETHICS STATEMENT

The study was approved by the National Board of Health Data (Region Zealand (REG‐059‐2021)). Ethical review and approval were waived for this study, as no human biological material was included in the project. The study was registered in the Danish Cancer Society Research Database (2019‐DCRC‐0066). We obtained informed consent from all participants in the study. Data were stored and protected following the guidelines of the Danish legislation regarding GDPR.

## Supporting information


Appendix S1.

## Data Availability

The supporting data are not publicly available due to research participant privacy restrictions. The data that support the findings of this study are available for collaborative research project upon reasonable request.
