# Supplementary material for: Social inequality in cancer survivorship: Educational differences in health‐related quality of life among 27,857 cancer survivors in Denmark
Source: Cancer Med. 2023 Sep 29;12(19):20150–62. doi: 10.1002/cam4.6596 (PMC10587985; doi:10.1002/cam4.6596)
Supplement: Supplementary file 1 — Appendix S1. [file CAM4-12-20150-s001.docx]

**Supplementary table 1.** Definition and categorization of physical, lifestyle and clinical variables

| **Information from questionnaire study** | | |
| --- | --- | --- |
| **Variable** | **Cancer type** | **Description and categorization** |
| Comorbidity | All | Study-specific list of common comorbid disorders included depression, anxiety, asthma, apoplexia, hypertension, migraine, arthritis, thyroid disorders, cardiovascular disease, osteoporosis, diabetes, impaired hearing, low vision/blindness, chronic obstructive pulmonary disease, and disorders of the nervous system (Parkinson’s disease, multiple sclerosis). Categorized as having 0, 1, or ≥2 comorbidities. |
| Body mass index | All | Body mass index (BMI) (kg/m^2^). Categorized as being underweight (<18.5), normal weight (18.5-24.9), overweight (25-29.9), or obese (≥30). |
| Smoking | All | Smoking was categorized as never, former, or current smoker. |
| Alcohol intake | All | Alcohol intake was defined as intake during the last week and categorized as no alcohol intake (0 units), alcohol intake within the recommended amount by the Danish health authorities at time of the study (1-7/14 units for women/men), and alcohol intake higher than the recommended amount (≥8/15 units for women/men). |
| **Information from clinical databases** | | |
| **Variable** | **Cancer type** | **Description and categorization** |
| Disease stage | Breast | Local/regional stage: any tumor size, any number of positive lymph nodes, and no distant metastases.  Advanced stage: distant metastases. |
|  | Prostate | Local/regional stage: TNM stage with any T, any N, and M0.  Advanced stage: TNM stage with distant metastases (M1). |
|  | Lung | Local/regional stage: TNM stage at IA, IB, IIA, IIB, or IIIA. Advanced stage: TNM stage IIIB, IIIC, IVA, or IVB. |
|  | Colon | Local/regional stage: UICC (8^th^ edition) stage I, II, or III. Advanced stage: UICC (8^th^ edition) stage IV. |
| Treatment | Breast | Curative: multimodality treatment for early-stage disease. Palliative: treatment for advanced stage disease. |
|  | Prostate | Curative: prostatectomy, active surveillance, or curatively intended radiotherapy. Palliative: palliative radiotherapy, endocrine therapy, or watchful waiting. |
|  | Lung | Curative: curatively intended chemo-, and/or radiotherapy, surgery or neo-/adjuvant therapy. Palliative: palliative chemo- and/or radiotherapy, and other treatment with palliative intent. |
|  | Colon | Curative: surgery with curative intent, with or without adjuvant chemotherapy.  Palliative: surgery with palliative intent and chemo-, and/or radiotherapy with palliative intent. |

**Supplementary table 2.** Differences in characteristics of respondents vs. non-respondents and individuals without digital mail

|  | **Comparison groups** | | | p-value |
| --- | --- | --- | --- | --- |
|  | Respondents  n=27,998 | Non-respondents  n=39,158 | Individuals without active digital mail  n=13,105 |  |
| Age at diagnosis, mean (sd) | 63.3 (9.1) | 64.9 (10.1) | 73.3 (8.8) | <0.0001 |
| Time since diagnosis, mean (sd) | 6.4 (2.8) | 6.4 (2.8) | 6.7 (2.9) | <0.0001 |
| Sex, n (%)  Male  Female | 12955 (47)  15043 (54) | 18723 (48)  20435 (52) | 5643 (43)  7462 (57) | <0.0001 |
| Cancer type, n (%)  Breast  Prostate  Lung  Colon | 11962 (43)  9633 (34)  1759 (6)  4644 (17) | 15891 (41)  14182 (36)  2910 (7)  6175 (16) | 4844 (37)  3729 (28)  1477 (11)  3055 (23) | <0.0001 |
| Educational level, n (%)  Short  Medium  Long  Missing | 2978 (11)  13941 (50)  10783 (38)  269 (1) | 7096 (18)  20805 (53)  10610 (27)  647 (2) | 5457 (42)  5892 (45)  1379 (11)  377 (3) | <0.0001 |

Respondents: cancer survivors who participated in the questionnaire study, Non-respondents: cancer survivors who received an invitation to participate in the questionnaire study, but did not respond; Individuals without active digital mail: cancer survivors who did not receive an invitation to participate in the questionnaire study, as they had actively closed their public digital mail-box. Significant difference in characteristics between groups were calculated using anova tests and chi-square tests.

**Supplementary table 3.** The association between educational level and the risk of reporting impaired functions or symptoms at clinical important levels among Danish cancer survivors, by cancer type and survival time

|  | **Education^a^** | **Breast** | | **Prostate** | | **Lung** | | **Colon** | |
| --- | --- | --- | --- | --- | --- | --- | --- | --- | --- |
|  |  | 2-5 years n=4269 | >5-12 years n=7599 | 2-5 years n=3524 | >5-12 years n=6082 | 2-5 years n=967 | >5-12 years n=787 | 2-5 years n=1882 | >5-12 years n=2747 |
|  |  | OR (99% CI) | OR (99% CI) | OR (99% CI) | OR (99% CI) | OR (99% CI) | OR (99% CI) | OR (99% CI) | OR (99% CI) |
| **Functioning scales (EORTC QLQ-C30)** | | | | | | | | | |
| Physical function | Short | 3.01 (2.22; 4.10) | 2.14 (1.70; 2.67) | 2.17 (1.57; 2.99) | 1.61 (1.25; 2.08) | 3.19 (1.82; 5.73) | 2.76 (1.47; 5.28) | 1.69 (1.08; 2.65) | 1.71 (1.18; 2.46) |
|  | Medium | 1.43 (1.19; 1.71) | 1.53 (1.34; 1.76) | 1.46 (1.17; 1.83) | 1.32 (1.12; 1.57) | 1.66 (1.11; 2.49) | 1.75 (1.12; 2.74) | 1.17 (0.87; 1.57) | 1.33 (1.03; 1.72) |
| Role function | Short | 2.86 (1.99; 4.07) | 2.42 (1.81; 3.21) | 2.04 (1.29; 3.17) | 1.94 (1.37; 2.73) | 2.69 (1.46; 5.02) | 2.08 (1.03; 4.21) | 2.37 (1.35; 4.13) | 1.71 (1.02; 2.83) |
|  | Medium | 1.28 (1.00; 1.64) | 1.64 (1.35; 1.99) | 1.57 (1.13; 2.19) | 1.48 (1.16; 1.90) | 1.71 (1.05; 2.88) | 1.80 (1.06; 3.16) | 1.07 (0.70; 1.64) | 1.48 (1.03; 2.15) |
| Emotional function | Short | 2.94 (1.94; 4.42) | 2.55 (1.79; 3.59) | 2.82 (1.43; 5.48) | 1.84 (1.09; 3.05) | 1.98 (0.93; 4.25) | 2.36 (0.75; 7.80) | 1.98 (0.87; 4.28) | 1.31 (0.57; 2.83) |
|  | Medium | 1.43 (1.07; 1.91) | 1.63 (1.28; 2.08) | 1.79 (1.08; 3.06) | 1.07 (0.74; 1.57) | 1.18 (0.64; 2.27) | 2.75 (1.16; 7.69) | 0.99 (0.56; 1.79) | 1.43 (0.85; 2.49) |
| Cognitive function | Short | 2.10 (1.52; 2.89) | 2.10 (1.64; 2.67) | 2.26 (1.54; 3.27) | 1.49 (1.10; 2.00) | 1.50 (0.82; 2.73) | 1.57 (0.79; 3.12) | 1.37 (0.80; 2.29) | 1.43 (0.93; 2.18) |
|  | Medium | 1.35 (1.12; 1.63) | 1.38 (1.19; 1.60) | 1.49 (1.14; 1.96) | 1.13 (0.92; 1.39) | 1.46 (0.93; 2.33) | 1.18 (0.71; 2.02) | 1.03 (0.73; 1.45) | 1.33 (0.99; 1.78) |
| Social function | Short | 1.84 (1.32; 2.55) | 1.79 (1.37; 2.31) | 1.14 (0.81; 1.60) | 1.14 (0.87; 1.50) | 1.51 (0.85; 2.71) | 1.42 (0.71; 2.83) | 1.42 (0.83; 2.37) | 1.19 (0.72; 1.90) |
|  | Medium | 1.18 (0.97; 1.44) | 1.42 (1.21; 1.67) | 1.11 (0.89; 1.38) | 1.23 (1.04; 1.45) | 1.16 (0.74; 1.83) | 1.40 (0.84; 2.38) | 0.87 (0.61; 1.25) | 1.33 (0.98; 1.84) |
| **Symptom scales and single-items (EORTC QLQ-C30)** | | | | | | | | | |
| Fatigue | Short | 2.48 (1.81; 3.41) | 2.02 (1.58; 2.57) | 2.17 (1.50; 3.13) | 1.44 (1.06; 1.96) | 2.15 (1.23; 3.77) | 1.75 (0.90; 3.38) | 1.55 (0.93; 2.55) | 1.36 (0.86; 2.11) |
|  | Medium | 1.29 (1.06; 1.57) | 1.37 (1.18; 1.60) | 1.40 (1.08; 1.84) | 1.15 (0.93; 1.41) | 1.36 (0.89; 2.12) | 1.73 (1.06; 2.88) | 0.83 (0.58; 1.18) | 1.30 (0.96; 1.77) |
| Nausea/  vomiting | Short | 2.34 (1.61; 3.37) | 1.79 (1.33; 2.39) | 1.80 (1.09; 2.91) | 1.34 (0.87; 2.01) | 1.39 (0.74; 2.59) | 2.27 (1.00; 5.20) | 1.52 (0.85; 2.64) | 1.62 (0.98; 2.62) |
|  | Medium | 1.30 (1.02; 1.67) | 1.40 (1.16; 1.69) | 1.35 (0.96; 1.93) | 1.23 (0.94; 1.62) | 1.05 (0.65; 1.73) | 1.94 (1.03; 3.86) | 0.87 (0.59; 1.29) | 1.41 (1.00; 2.01) |
| Pain | Short | 2.83 (2.08; 3.86) | 2.15 (1.72; 2.70) | 2.34 (1.67; 3.27) | 1.62 (1.23; 2.12) | 2.22 (1.27; 3.90) | 1.92 (1.01; 3.65) | 1.84 (1.15; 2.94) | 1.67 (1.13; 2.44) |
|  | Medium | 1.59 (1.33; 1.90) | 1.55 (1.36; 1.78) | 1.51 (1.19; 1.92) | 1.38 (1.16; 1.65) | 1.37 (0.89; 2.13) | 1.51 (0.93; 2.48) | 1.14 (0.83; 1.57) | 1.46 (1.12; 1.91) |
| Dyspnea | Short | 2.64 (1.94; 3.61) | 1.70 (1.34; 2.14) | 2.14 (1.55; 2.93) | 1.80 (1.40; 2.32) | 2.06 (1.13; 3.90) | 2.25 (1.12; 4.74) | 1.79 (1.15; 2.79) | 1.53 (1.06; 2.19) |
|  | Medium | 1.64 (1.36; 1.98) | 1.39 (1.21; 1.60) | 1.67 (1.35; 2.08) | 1.55 (1.31; 1.83) | 1.47 (0.96; 2.26) | 1.45 (0.90; 2.32) | 1.19 (0.89; 1.60) | 1.43 (1.11; 1.84) |
| Insomnia | Short | 1.80 (1.29; 2.48) | 1.96 (1.54; 2.49) | 1.29 (0.80; 2.01) | 1.39 (0.96; 1.98) | 1.62 (0.87; 3.01) | 1.69 (0.79; 3.59) | 1.22 (0.68; 2.12) | 1.04 (0.64; 1.67) |
|  | Medium | 1.25 (1.03; 1.52) | 1.30 (1.12; 1.51) | 1.38 (1.03; 1.87) | 1.22 (0.97; 1.55) | 1.00 (0.62; 1.66) | 1.65 (0.94; 2.99) | 0.79 (0.54; 1.16) | 1.09 (0.79; 1.50) |
| Appetite loss | Short | 3.38 (1.66; 6.69) | 3.22 (1.88; 5.40) | 1.87 (0.79; 4.19) | 2.15 (1.10; 4.06) | 2.20 (0.90; 5.48) | 3.76 (1.13; 14.50) | 1.63 (0.57; 4.27) | 1.37 (0.53; 3.24) |
|  | Medium | 1.94 (1.17; 3.32) | 1.91 (1.30; 2.87) | 1.40 (0.77; 2.65) | 1.47 (0.91; 2.44) | 1.64 (0.81; 3.64) | 2.29 (0.84; 7.99) | 1.06 (0.52; 2.27) | 1.29 (0.70; 2.46) |
| Constipation | Short | 1.40 (0.78; 2.40) | 1.97 (1.31; 2.91) | 2.08 (1.00; 4.18) | 1.09 (0.58; 1.96) | 1.08 (0.41; 2.72) | 1.47 (0.44; 4.72) | 2.01 (0.92; 4.22) | 1.07 (0.51; 2.11) |
|  | Medium | 1.15 (0.81; 1.62) | 1.28 (0.98; 1.69) | 1.64 (0.97; 2.84) | 1.19 (0.81; 1.77) | 0.98 (0.49; 2.08) | 1.60 (0.70; 4.18) | 1.30 (0.76; 2.29) | 1.22 (0.77; 1.97) |
| Diarrhea | Short | 1.81 (1.27; 2.56) | 1.24 (0.92; 1.64) | 1.59 (1.10; 2.27) | 1.32 (0.98; 1.77) | 1.66 (0.91; 3.01) | 1.19 (0.58; 2.42) | 1.19 (0.76; 1.84) | 1.05 (0.73; 1.49) |
|  | Medium | 1.16 (0.93; 1.44) | 1.25 (1.06; 1.49) | 1.51 (1.19; 1.93) | 1.45 (1.20; 1.75) | 1.13 (0.72; 1.83) | 1.19 (0.71; 2.05) | 1.22 (0.93; 1.61) | 1.11 (0.88; 1.40) |
| Financial difficulties | Short | 2.45 (1.67; 3.57) | 2.12 (1.56; 2.85) | 3.06 (1.80; 5.17) | 1.99 (1.30; 3.01) | 2.80 (1.38; 5.76) | 1.91 (0.84; 4.33) | 2.07 (1.08; 3.84) | 1.73 (0.93; 3.10) |
|  | Medium | 1.34 (1.04; 1.71) | 1.45 (1.19; 1.76) | 2.03 (1.37; 3.08) | 1.82 (1.37; 2.43) | 1.80 (1.01; 3.34) | 1.60 (0.86; 3.12) | 0.98 (0.62; 1.57) | 1.39 (0.91; 2.15) |

Long education is the reference for all analyses. All analyses are adjusted for age at diagnosis, sex and time since diagnosis. OR, odds ratio; CI, confidence interval; EORTC QLQ-C30, European Organization for Research and Treatment of Cancer Quality of Life Core Questionnaire.

^a^ Education: categorized as short (mandatory school; ≤ 9 years), medium (secondary education or vocational education; 10-12 years) and long education (higher education; > 12 years).

**Supplementary table 4.** The association between educational level and the risk of reporting impaired functioning or symptoms at clinically important levels in Danish breast, prostate, lung, and colon cancer survivors with adjustment for cohabitation, urbanicity, lifestyle, and clinical factors

|  | **Education^a^** | **Cancer survivors** | | | |
| --- | --- | --- | --- | --- | --- |
|  |  | **Breast** | **Prostate** | **Lung** | **Colon** |
|  |  | OR (99 % CI) | OR (99 % CI) | OR (99 % CI) | OR (99 % CI) |
| **Functioning scales (EORTC QLQ-C30)** | | | | | |
| Physical function | Short | 1.71 (1.36; 2.15) | 1.42 (1.05; 1.91) | 2.67 (1.55; 4.69) | 1.31 (0.90; 1.88) |
|  | Medium | 1.26 (1.10; 1.44) | 1.20 (0.98; 1.47) | 1.61 (1.09; 2.37) | 1.13 (0.88; 1.44) |
| Role function | Short | 1.98 (1.48; 2.63) | 1.73 (1.15; 2.58) | 2.30 (1.29; 4.11) | 1.39 (0.85; 2.22) |
|  | Medium | 1.38 (1.14; 1.67) | 1.42 (1.06; 1.90) | 1.78 (1.13; 2.85) | 1.18 (0.84; 1.66) |
| Emotional function | Short | 2.00 (1.41; 2.79) | 1.77 (0.96; 3.20) | 1.94 (0.85; 4.44) | 1.21 (0.56; 2.47) |
|  | Medium | 1.42 (1.13; 1.79) | 1.10 (0.71; 1.73) | 1.81 (0.97; 3.60) | 1.16 (0.72; 1.90) |
| Cognitive function | Short | 1.84 (1.45; 2.33) | 1.50 (1.06; 2.11) | 1.30 (0.73; 2.28) | 1.28 (0.84; 1.93) |
|  | Medium | 1.24 (1.07; 1.43) | 1.21 (0.96; 1.53) | 1.21 (0.79; 1.86) | 1.19 (0.91; 1.56) |
| Social function | Short | 1.40 (1.08; 1.80) | 1.03 (0.76; 1.38) | 1.19 (0.67; 2.08) | 1.01 (0.64; 1.57) |
|  | Medium | 1.19 (1.02; 1.38) | 1.09 (0.90; 1.32) | 1.14 (0.75; 1.75) | 1.05 (0.79; 1.40) |
| **Symptom scales and single-items (EORTC QLQ-C30)** | | | | | |
| Fatigue | Short | 1.64 (1.29; 2.10) | 1.61 (1.13; 2.27) | 1.54 (0.89; 2.64) | 1.19 (0.77; 1.82) |
|  | Medium | 1.23 (1.06; 1.43) | 1.17 (0.92; 1.49) | 1.43 (0.95; 2.17) | 1.07 (0.80; 1.43) |
| Nausea/vomiting | Short | 1.69 (1.27; 2.23) | 1.33 (0.83; 2.08) | 1.74 (0.92; 3.32) | 1.62 (1.01; 2.54) |
|  | Medium | 1.28 (1.07; 1.54) | 1.20 (0.89; 1.64) | 1.50 (0.92; 2.52) | 1.27 (0.92; 1.75) |
| Pain | Short | 1.86 (1.49; 2.33) | 1.75 (1.29; 2.36) | 1.82 (1.07; 3.08) | 1.36 (0.93; 1.96) |
|  | Medium | 1.35 (1.18; 1.54) | 1.29 (1.05; 1.59) | 1.38 (0.93; 2.07) | 1.19 (0.92; 1.52) |
| Dyspnea | Short | 1.57 (1.25; 1.98) | 1.76 (1.32; 2.35) | 1.71 (0.95; 3.16) | 1.51 (1.06; 2.16) |
|  | Medium | 1.24 (1.08; 1.43) | 1.46 (1.21; 1.77) | 1.22 (0.81; 1.82) | 1.30 (1.02; 1.65) |
| Insomnia | Short | 1.80 (1.43; 2.27) | 1.38 (0.92; 2.06) | 2.17 (1.18; 4.00) | 1.08 (0.68; 1.68) |
|  | Medium | 1.22 (1.06; 1.41) | 1.29 (0.99; 1.70) | 1.53 (0.95; 2.50) | 0.88 (0.66; 1.19) |
| Appetite loss | Short | 3.04 (1.80; 5.08) | 1.97 (0.96; 3.93) | 1.91 (0.76; 4.88) | 1.31 (0.53; 3.01) |
|  | Medium | 1.70 (1.16; 2.54) | 1.40 (0.84; 2.40) | 1.60 (0.78; 3.52) | 1.18 (0.66; 2.13) |
| Constipation | Short | 1.63 (1.10; 2.39) | 1.20 (0.59; 2.32) | 1.57 (0.59; 4.19) | 1.28 (0.66; 2.38) |
|  | Medium | 1.19 (0.92; 1.54) | 1.39 (0.88; 2.24) | 1.83 (0.88; 4.18) | 1.23 (0.80; 1.91) |
| Diarrhea | Short | 1.26 (0.96; 1.64) | 1.32 (0.94; 1.83) | 1.53 (0.87; 2.70) | 1.11 (0.79; 1.55) |
|  | Medium | 1.15 (0.98; 1.35) | 1.44 (1.16; 1.78) | 1.15 (0.75; 1.79) | 1.15 (0.93; 1.43) |
| Financial difficulties | Short | 1.77 (1.31; 2.37) | 1.92 (1.21; 3.02) | 2.43 (1.24; 4.84) | 1.79 (1.03; 3.07) |
|  | Medium | 1.26 (1.04; 1.53) | 1.53 (1.11; 2.13) | 1.56 (0.91; 2.77) | 1.30 (0.89; 1.94) |

Long education is the reference for all analyses. All analyses are adjusted for age at diagnosis, sex, time since diagnosis, cohabitation, urbanicity, comorbidity, stage at diagnosis, alcohol, smoking, BMI, and treatment. OR, odds ratio; CI, confidence interval; EORTC QLQ-C30, European Organization for Research and Treatment of Cancer Quality of Life Core Questionnaire; BMI, body mass index.

^a^ Education: categorized as short (mandatory school; ≤ 9 years), medium (secondary education or vocational education; 10-12 years), and long education (higher education; > 12 years).

**
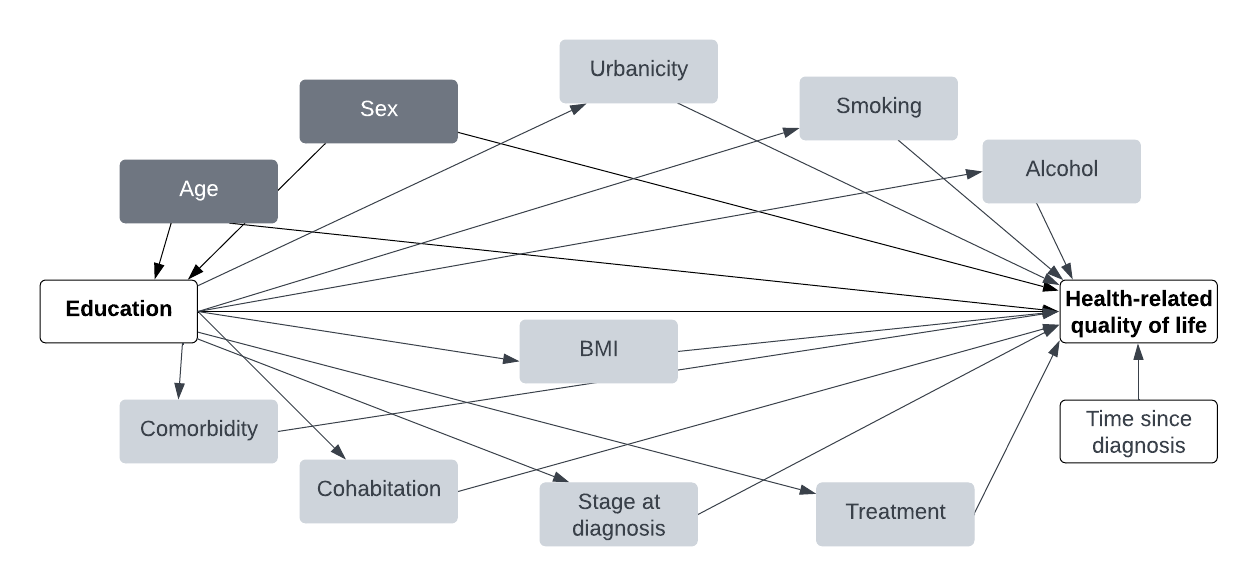
**

**Supplementary figure 1.** Directed acyclic graph for the hypothesized association between educational level and HRQOL. We did not include arrows for associations between confounders (age, sex) and mediators (comorbidity, cohabitation, urbanicity, stage at diagnosis, treatment, BMI, smoking, alcohol). HRQOL, health-related quality of life; BMI, body mass index
